# Central neuronal functions of histamine H_4_ receptors

**DOI:** 10.18632/oncotarget.15289

**Published:** 2017-02-11

**Authors:** Maria Domenica Sanna, Nicoletta Galeotti

**Affiliations:** Department of Neuroscience, Psychology, Drug Research and Child Health, Section of Pharmacology, University of Florence, Florence, Italy

**Keywords:** histamine H4 receptors, central nervous system, pain, anxiety, memory, Neuroscience

The human histamine H_4_ receptor (H_4_R), the most recently discovered histamine receptor, is predominantly expressed on inflammatory cells and lymphoid tissues and characterized as the immune system histamine receptor with a pro-inflammatory role. The occurrence of H_4_R on human and rodent neurons has also been recently reported, but, conversely to the well-characterized immunological role of the peripheral H_4_R, much less is known about its functions in the central nervous system (CNS).

The expression and neuronal role of H_4_R has been controversially discussed since its discovery. The human and rat H_4_R mRNA and protein were detected in dorsal root ganglia (DRG), spinal cord and several brain regions [1, 2], but the difficulty to generate H_4_R antibodies with high specificity generates results that should be interpreted with some caution. Some indication for a functional role in the CNS for this receptor came from studies with selective ligands [3, 4]. However, in the absence of knockout controls, off-target effects of the H_4_R ligands cannot be fully excluded. Although H_4_R-deficient mice were generated more than a decade ago, they have not been used to elucidate the physiopathological role of this receptor subtype in the CNS. The study of Sanna et al. [5] is the first investigation on the functional role of neuronal H_4_R through the characterization of the behavioural phenotype of H_4_R-deficient mice.

Authors [6] showed that a prominent role of neuronal H_4_R appears to be the modulation of pain perception. In human and rodents, expression of H_4_R mRNA is the highest in the spinal cord and H_4_R protein is strongly expressed in sensory neurons of DRG and in the lumbar spinal cord, especially laminae I and II [1, 2], consistent with a role of H_4_R in pain perception. Conversely to what expected from the pro-nociceptive profile obtained by peripheral H_4_R stimulation, H_4_R-deficient mice had unaltered sensitivity to thermal and mechanical stimuli, and showed increased pain hypersensitivity in the presence of a peripheral neuropathy. This hypernociceptive phenotype indicates an involvement of neuronal H_4_R in the response to a condition of pathological chronic pain of neuronal origin rather than in the regulation of the physiological maintenance of the pain threshold. A dual role of neuronal and peripheral H_4_R on pain processes can be postulated: pain-reducing activity by neuronal H_4_R stimulation, pain-promoting pro-inflammatory effects by peripheral H_4_R stimulation.

A role of the histaminergic system in the modulation of anxiety has been suggested, but investigations produced contradictory results and opposite roles for histamine H_1_R, H_2_R and H_3_R subtypes have been reported [6]. H_4_R deficiency exacerbated the response to an anxiety-provoking environment. This anxiogenic-like phenotype of H_4_R-deficient mice indicates a positive effect of neuronal H_4_R stimulation on anxiety management. This hypothesis was supported by the anxiolytic-like effects produced by centrally administered H_4_R agonists in behavioural tasks in mice [4].

Brain histamine plays a fundamental role in eating behaviour and induces loss of appetite and suppression of food intake mainly via H_1_R [6]. H_4_R-deficient mice showed an increase in food intake, consistent with the reduction of food consumption observed after intracerebroventricular administration of the H_4_R full agonist VUF 8430. Neuronal H_4_R might, thus, exert a synergic action with H_1_R in the histaminergic regulation of eating behaviour.

The histaminergic system is known to be involved in the regulation of locomotor activity. Histidine decarboxylase, H_1_R, H_2_R and H_3_R knockout mice showed reduced locomotor activity [7]. Conversely, behavioural characterization of H_4_R-deficient mice showed an increase in ambulation in an open field and in exploratory activity, revealing an increased spontaneous locomotor activity. These findings highlight a role of H_4_R on motor behaviour at odds with that observed for the other histamine receptor subtypes.

The study of Sanna et al. [6] illustrated a peculiar and selective behavioural profile of H_4_R-deficient mice with a modest or absent involvement of H_4_R in some of neuronal functions modulated by the histaminergic system, such as depression and memory. Pharmacological or genetic loss of histamine or histamine receptor function in animals produces phenotypes that model human depression [6]. H_4_R-deficient mice had higher immobility time than wild type mice in a behavioural despair paradigm, showing a depressant-like response. However, in the same testing environment, central administration of H_4_R agonists was devoid of any antidepressant-like activity, suggesting a minor role of H_4_R in depression. Authors observed that H_4_R deficiency induces an anxiogenic-like behaviour and the increased immobility time recorded might be a response to an anxiety-promoting environment rather than a depressant-like response. Findings on H_4_R-deficient mice also exclude an outstanding role of H_4_R in the histaminergic modulation of memory processes. Although histaminergic neurons project to regions important for cognitive functions, H_4_R-deficient mice did not show any alteration of both working and recognition memory.

Characterization of the behavioural phenotype of H_4_R-deficient mice assessed a functional role of neuronal H_4_R and highlighted the importance of its integrity in the histaminergic regulation of important neurophysiological functions, including pain sensitivity and anxiety. Since the identification of H_4_R, several ligands activating this receptor have been described and more compounds are in development. Targeting neuronal H_4_R with selective agonists might be a clinical relevant therapeutic approach to be exploited to relieve neuropathic pain and to better manage anxiety and anxiety-related disorders.
